# Therapeutic Potential of *Ranunculus* Species (Ranunculaceae): A Literature Review on Traditional Medicinal Herbs

**DOI:** 10.3390/plants11121599

**Published:** 2022-06-17

**Authors:** Youn-Kyoung Goo

**Affiliations:** Department of Parasitology and Tropical Medicine, School of Medicine, Kyungpook National University, Daegu 41944, Korea; kuku1819@knu.ac.kr

**Keywords:** ethnopharmacologic effect, herbal medicine, *Ranunculus* species

## Abstract

The genus *Ranunculus* includes approximately 600 species and is distributed worldwide. To date, several researchers have investigated the chemical and biological activities of *Ranunculus* species, and my research team has found them to have antimalarial effects. This review is based on the available information on the traditional uses and pharmacological studies of *Ranunculus* species. The present paper covers online literature, particularly from 2010 to 2021, and books on the ethnopharmacology and botany of *Ranunculus* species. Previous studies on the biological activity of crude or purified compounds from *Ranunculus* species, including *R. sceleratus* Linn., *R. japonicus* Thunb., *R. muricatus* Linn., *R. ternatus* Thunb., *R. arvensis* Linn., *R. diffusus* DC., *R. sardous* Crantz, *R. ficaria* Linn., *R. hyperboreus* Rotlb., and *R. pedatus* Waldst. & Kit., have provided new insights into their activities, such as antibacterial and antiprotozoal effects as well as antioxidant, immunomodulatory, and anticarcinogenic properties. In addition, the anti-inflammatory and analgesic effects of plants used in traditional medicine applications have been confirmed. Therefore, there is a need for more diverse studies on the chemical and pharmacological activities of highly purified molecules from *Ranunculus* species extracts to understand the mechanisms underlying their activities and identify novel drug candidates.

## 1. Introduction

The genus *Ranunculus* includes approximately 600 species globally. Recent taxonomic reports suggest that this genus has a monophyletic origin and is divided into two subgenera and seventeen sections [1]. Owing to its wide distribution, the genus has high genetic diversity. Several *Ranunculus* species have been used in folk medicine to treat various diseases or symptoms, such as jaundice, nebula, edema, malaria, asthma, pain, gout, rheumatism, inflammatory skin disorders, cancer, and hypertension. In addition, researchers have reported that *Ranunculus* extracts possess antioxidant, anti-inflammatory, antimutagenic, antimalarial, antibacterial, antitumoral, cardioprotective, and wound-healing properties [2,3,4,5,6,7].

Over the last decade, various studies have investigated the chemical components and pharmacological activities of *Ranunculus* species [8,9]. However, no recent review has been published detailing the aspects of the plants that have been investigated, including their biology, traditional uses, phytoconstituents, therapeutic activities, and clinical applications, since a previous review article was reported in 2012 [10]. Thus, this article aims to provide an up-to-date survey of the advances in and prospects of the research on the phytochemicals and pharmacological potential of *Ranunculus* species.

## 2. Search Strategy

This review article is based on the information available on the phytochemical, toxicological, and pharmacological studies on the traditional uses of *Ranunculus* species. The present paper covers online literature (Google Scholar, PubMed, ScienceDirect, Scopus, SpringerLink, and Web of Science), particularly from 2010 to 2021, and books on the ethnopharmacology and botany of *Ranunculus* species. The following words were used as key search terms: (“*Ranunculus*” OR “*Ranunculus* species”) AND (“herbal medicine” OR “herb medicine” OR “ethnopharmacological effects” OR “ethnopharmacological activity” OR “phytomedicine” OR “treatment” OR “drug”. The range of the article publication year for the search (from 2010 to 2021) was selected because the previous review by Aslam et al. covered almost all literature data published by 2012 [10].

## 3. Taxonomy, Distribution, and Morphology

Ranunculaceae Juss., or the buttercup family, has a worldwide distribution, representing a large group comprising more than 2500 species belonging to 59 genera. Its family members live under a wide range of ecological conditions, especially in the Northern Hemisphere [1,11]. Among the family, *Ranunculus*, comprising 600 species, is distributed across all continents [11]. *Ranunculus* species are highly genetically diverse; therefore, their classification is challenging. As a result, generic delimitation and infrageneric classification of these species are still under consideration.

Initially, *Ranunculus* species were classified based on the descriptions of their achenes (e.g., the shape of their body and beak, pericarp structure, and indumentum), flowers (e.g., the number of sepals and honey-leaves, gloss and color of the petals, and shape of the nectaries), roots (e.g., whether they were uniform or dimorphic with fibrous and tuberous roots) [12], and fruit anatomy [13]. Later, Tamura classified the genera into seven subgenera based on the reassessment of the achene structure: *Pallasiantha*, *Coptidium*, *Ficaria*, *Batrachium*, *Crymodes*, *Gampsoceras*, and *Ranunculus* [1,11]. In this classification, the subgenera of *Ranunculus* were further subdivided into 20 sections [11].

Subsequently, DNA markers were utilized to delineate the phylogenetic relationships within Ranunculaceae [14,15,16,17,18,19,20,21,22,23]. The sequences of the internal transcribed spacer region of nuclear ribosomal DNA are mostly used as DNA barcode markers for phylogenetic studies at the generic/subgeneric level [24,25]. In combination with data from the chloroplast genome and other external data, this nuclear marker also offers insights into the reticulate patterns caused by hybridization [26,27]. Moreover, a complete study of the taxonomy of the genus using both DNA markers and morphological data suggested the separation of 226 species into two subgenera and 17 sections [20].

## 4. Phytochemical Investigations of *Ranunculus* Species

*Ranunculus sceleratus* Linn., commonly known as the celery-leaved buttercup, is a flowering plant species distributed over the Northern Hemisphere. The main constituents of *R. sceleratus* L. are flavonoids, steroids such as pyrogallol tannins, and the glycoside ranunculin [28]. Ranunculin is hydrolyzed after the leaves of *R. sceleratus* L. are dried or crushed and generates protoanemonin associated with the toxic properties of buttercups. Because of its instability, protoanemonin dimerizes to produce anemonin, a nonirritant form [29,30]. In addition, the 70% ethanolic extracts from the aerial parts of *R. sceleratus* L. have been found to be abundant in myristic acid [31], and sapigenin 4′-*O*-alpha-rhamnopyranoside, apigenin 7-*O*-beta-glucopyranosyl-4′-*O*-alpha-rhamnopyranoside, tricin 7-*O*-beta-glucopyranoside, tricin, and isoscopoletin have been identified as *R. sceleratus*-derived compounds in the extract [32].

*Ranunculus ficaria* Linn. is known as lesser celandine. The compositions found in *R. ficaria* L. were ranunculin and its enzymatic reaction products, flavonoids such as quercetin and rutoside, saponosides with hederagenin, oleanolic acid aglyca, macerate, and tinctures [33,34,35].

The components of *R. japonicus* Thunb. revealed by a Waters Acquity Ultra Performance liquid chromatography system were lactone glycosides, flavonoid glycosides, and aglycones including ranunculin, tricin, adonivernite, orientin, isorientin, vitexin, 6-C-β-D-glucosyl-8-C-α-L-arabinosylapigenin, and tricin-7-O-β-D-glucopyranoside [36].

*Ranunculus muricatus* Linn. is also known as spiny fruit buttercup. Phytochemical analysis of *R. muricatus* L. revealed the presence of saponins, tannins, phenols, flavonoids, alkaloids, cardiac glycosides, anthocyanins, carbohydrates, coumarins, and phytosterols [8,37,38]. The major constituents by HPLC were stigmast-4-ene-3,6-dione, stigmasterol, anemonin, β-sitosterol, protocatechuic aldehyde, protocatechuic acid, lutein, flavonoid glycosides, ranunculoside A, ranunculoside B, and ranunculone C, in addition to two potent antioxidants, caffeoyl-β-*D*-glucopyranoside, and 1,3-dihydroxy-2-tetracosanoylamino-4-(E)-nonadecene [9,39,40,41]. Moreover, four compounds, muriolide, muricazine, chalcone 4-benzyloxylonchocarpin, and new-to-nature anthraquinone muracatanes B, were recently isolated [42,43].

Phytochemical analyses of *R. ternatus* Thunb. reported that the plant contains flavonoids, glycosides, benzine, organic acids, sterols, esters, amino acids, and constant and trace elements [44]. Furthermore, *R. ternatus* ethyl acetate extract constitutes contain sternbin, methylparaben, 3-[(4-O-d-glucopyranosyl)-phenyl]-2-propenoic acid, linocaffein, β-d-glucose, robustaflavone-4′-methylether, kayaflavone, podocarpus flavone A, bilobetin, isoginkgetin, amentoflavone, ternatoside A, ternatoside B, and 4-*O*-d-glucopyranosyl-p-coumaric acid [45,46,47]. Furthermore, methyl (R)-3-[2-(3,4-dihydroxybenzoyl)-4,5-dihydroxyphenyl]-2-hydroxypropanoate was isolated from *R. ternatus* roots [48].

*Ranunculus arvensis* Linn. is commonly known as field buttercup. Phytochemical analysis indicated that *R. arvensis* L. possesses rutin, caffeic acids, and classes of flavonoids and phenolics, including flavonol glycosides of quercetin, kaempferol, isorhamnetin, and their aglycons [49].

In *Ranunculus* species, several bioactive compounds and *Ranunculus*-specific constituents have been identified, such as ranunculosides, muricazine, and muracatanes. Although many other species related to *Ranunculus* have also been studied to evaluate their pharmacological activities, the novel bioactive compounds found in *Ranunculus* species with high pharmacological effects show nutraceutical and pharmaceutical potential. Pharmacological properties and molecular formula of *Ranunculus* species compounds reported in articles published from 2010 to 2021 are summarized in Table 1.

## 5. Pharmacological Activities of *Ranunculus* Species

### 5.1. Ranunculus sceleratus Linn.

All parts of *Ranunculus sceleratus* Linn. are poisonous when fresh; however, the plant is used in folk medicine to treat various diseases after heating or drying [7]. In recent decades, ethnopharmacological effects have been experimentally proven by several studies (Table 2). The two ranunculins, protoanemonin and anemonin, have shown fungicidal, antimicrobial, antimutanenic, and antipyretic properties [29,30,51], and have been used for ethnopharmacological purposes in many countries [52,53]. Sharif et al. performed an in vivo study to evaluate the effects of hypertension treatment using normotensive and fructose-induced hypertensive rats, in which the aqueous fraction produced the most interesting effects. Furthermore, mechanistic studies with various pharmacological antagonists have demonstrated that the hypotensive response induced by *R. sceleratus* L. is caused by the involvement of a muscarinic receptor, angiotensin-converting enzyme inhibition, ganglionic block, and nitric oxide release [54]. In addition, the 70% ethanolic extracts from the aerial parts of *R. sceleratus* L. revealed that abundant myristic acid in the extract inhibited nitrite concentration in LPS-stimulated RAW 264.7 macrophage cell line [31]. Moreover, *R. sceleratus*-derived compounds, sapigenin 4′-O-alpha-rhamnopyranoside, apigenin 7-O-beta-glucopyranosyl-4′-O-alpha-rhamnopyranoside, tricin 7-O-beta-glucopyranoside, tricin, and isoscopoletin, showed inhibitory activity against the hepatitis B virus [32]. In addition to the treatment effect of R. sceleratus extract, fresh *R. sceleratus* for TianJiu therapy, which involves adding Chinese medicinal herbal paste on designated acupoints, showed good therapeutic effect on intrahepatic cholestasis in rats, although the fresh form of *R. sceleratus* L. is known as an irritant [55]. Specific mechanisms by which the extract induces irritant or nonirritant responses have not been revealed. To eluciate this phenomenon, a methanolic extract of *R. sceleratus* L. was used to demonstrate the mechanism of both irritant and non-irritant properties induced by the extract in topical inflammation. When arachidonic acid elicited the inflammatory process, the effect of the extract was generally proinflammatory or neutral. However, if the response was caused by the application of an irritant, such as etradecanoylphorbol acetate, the extract mainly resulted in anti-inflammatory effects. This effect was mentioned as a counter-irritant, and the extract itself could be an irritant in physiological conditions but could also counteract the action of previously applied irritants [7].

### 5.2. Ranunculus ficaria Linn.

*Ranunculus ficaria* Linn. is an herbal astringent commonly used to treat hemorrhoids internally or externally [67]. Various methods have been applied for ethnopharmacological use. Infusion or decoction of the leaves and roots of *R. ficaria* was known to have trophic and anti-inflammatory effects in varicose veins, hemorrhoids, and skin disorders in Romania. The macerate and tinctures obtained from this plant are used to treat hemorrhoids by stimulating blood circulation as a traditional medication [67]. The compositions found in *R. ficaria* could inhibit nitrite accumulation, and thus may be useful for preventing inflammatory diseases mediated by the excessive production of nitric oxide, according to an in vitro macrophage study. However, a previous report suggested that clinicians should consider using lesser celandine (pilewort, *R. ficaria*) as a causative agent owing to its hepatotoxicity [35,68].

### 5.3. Ranunculus japonicus Thunb.

*Ranunculus japonicus* Thunb. has been used to treat malaria, jaundice, migraines, stomachaches, arthralgia, crane-like arthropathy, ulcers, toothaches, and eye inflammation since Zhou Hou Bei Ji Fang was first recorded more than 1800 years ago [69]. Since then, studies have demonstrated various phytomedicinal activities, such as the protective effect on heart diseases including myocardial ischemic-reperfusion injury, hypertrophy in cardiomyocytes, and high blood pressure by alleviating chronic [Ca^2+^] *i* overload, as well as therapeutic effects on rheumatoid arthritis and decreasing intracellular [Ca^2+^] *i* in vascular smooth muscle cells [50,57,58]. In addition, *R. japonicus* extracts showed antimalarial effects in in vitro culture of *Plasmodium falciparum* and in vivo rodent malaria experimental systems of *Plasmodium berghei* [56].

### 5.4. Ranunculus muricatus Linn.

*Ranunculus muricatus* Linn. has tremendous medicinal potentials [70]. It is used by the local population as a folk medicine for cough, asthma, heart disease, jaundice, diarrhea, dysentery, urinary infection, eczema, lymphatic tuberculosis, dental diseases, ringworm infection, and leprosy [71,72]. In addition, it exhibits antioxidant, anti-inflammatory, antibacterial, antifungal, analgesic, and cytotoxic activities [37,59,60,73]. Therefore, among *Ranunculus* species, *R. muricatus* L. is the most extensively studied. Several constituents identified in *R. muricatus* L. exhibit phytochemical activities. For example, the major isolated constituents are stigmast-4-ene-3,6-dione, stigmasterol, anemonin, β-sitosterol, protocatechuic aldehyde, protocatechuic acid, lutein, flavonoid glycosides, ranunculoside A, ranunculoside B, and ranunculone C, in addition to the two potent antioxidants, caffeoyl-β-*D*-glucopyranoside and 1,3-dihydroxy-2-tetracosanoylamino-4-(E)-nonadecene [9,39,40,41]. Moreover, two recently isolated compounds, muriolide, a new lactone, and muricazine, a new hydrazine derivative, exhibited robust free radical scavenging properties and exerted an inhibitory effect on lipoxygenase [42]. Finally, chalcone 4-benzyloxylonchocarpin, which inhibits AcheE, and the new-to-nature anthraquinone muracatanes B, which inhibits α-glucosidase, were isolated from *R. muricatus* L. [43].

### 5.5. Ranunculus diffusus DC.

The phytomedicinal effects of *Ranunculus diffusus* DC. have recently been reported. The methanol extract of *R. diffusus* showed photoaging protective effects on ultraviolet B radiation-induced skin by inhibiting the p38-AP-1 signal cascade. In addition, the extract exerted anti-inflammatory effects without toxicity by suppressing Src and Syk, which are targets of NF-κB signaling [63,64].

### 5.6. Ranunculus ternatus Thunb.

*Ranunculus ternatus* Thunb. has been used in traditional Chinese medicine [74] because of its effects on malignant lymphoma, leukemia, pulmonary tuberculosis, breast tumors, goiters, esophageal tumors, lung disease, gastric problems, and other health conditions [75,76,77]. Constituents of *R. ternatus*, such as amentoflavone and podocarpus flavone A, induce apoptosis [78,79]; however, their mechanisms have not been evaluated. Furthermore, n-butyl-β-D-fructofuranoside, isolated from *R. ternatus* roots, demonstrated significant therapeutic activity against tuberculosis [48]. Finally, the ethyl acetate extract of *R. ternatus* exerts caspase-7-dependent apoptosis in a cancer model [61].

### 5.7. Ranunculus arvensis Linn.

*Ranunculus arvensis* Linn. has been widely used to treat arthritis, asthma, hay fever, rheumatism, psoriasis, gut diseases, and rheumatic diseases [49]. Moreover, *R. arvensis* extracts showed antioxidant and anticarcinogenic activities [49,62]. However, topical use of the plant may cause contact dermatitis, such as skin inflammation, skin burns, and injury of mucous membranes [80,81,82].

### 5.8. Ranunculus hyperboreus Rotlb.

*Ranunculus hyperboreus* Rotlb. is a subarctic and subalpine plant that lives in extreme environmental conditions. *R. hyperboreus* extract induces anti-inflammatory activity by regulating the gene expression and protein levels of inflammation-related enzymes, such as iNOS and COX-2, and proinflammatory cytokines, such as TNF-α, IL-1β, and IL-6 [65].

### 5.9. Ranunculus pedatus Waldst. & Kit.

The wound healing activity of *Ranunculus pedatus* Waldst. & Kitt. was evaluated using its methanolic extract and was found to exert significant effects on wound healing with robust anti-inflammatory activity in both incision and excision wound animal models [66].

## 6. Conclusions

The chemical and biological activities of *Ranunculus* species have been investigated using plant extracts. Contemporary research on the biological activity of the extracts of the species mentioned above has uncovered many activities, including antibacterial, antiviral, and antiprotozoal effects, as well as antioxidant and anticarcinogenic properties. In addition, these studies have demonstrated that herbal extracts exert hepatoprotective, hypoglycemic, and thyroid regulatory effects. Moreover, the anti-inflammatory and analgesic effects of the plants, known from the application of traditional medicine, have been confirmed. Furthermore, the molecules isolated from *Ranunculus* species showed promising pharmacological activity. Therefore, it is expected that effective purified molecules could be discovered from *Ranunculus* species to develop novel drugs through intensive research.

## Figures and Tables

**Table 1:** Pharmacological properties and molecular formula of *Ranunculus* species compounds reported in articles published from 2010 to 2021.

*Ranunculus* Species	Molecule	Molecular Formula	Pharmacological Activity	Ref
*Ranunculus japonicus* Thunb.	berberine	C_20_H_18_NO_4_^+^	inhibited the migration capacity of RA-FLSs in a dose-dependent manner	[50]
	yangonin	C_15_H_14_O_4_		
*Ranunculus muricatus* Linn.	muricazine	C_16_H_10_N_2_O_4_	antioxidant effect, lipoxygenase, and urease inhibitory activities.	[42]
	4-benzyloxylonchocarpin	C_27_H_24_O_4_	acetylcholinesterase inhibitory effect	[43]
	muracatane B	C_14_H_8_O_5_	alpha-glucosidase inhibitory effect	[43]
	4-methoxylonchocarpin	C_21_H_20_O_4_	moderate cytotoxic effects towards ovarian carcinoma, colorectal adenocarcinoma, breast cancer, and thyroid carcinoma	[43]
	muriolide	C_15_H_15_O_8_	antioxidant and lipoxygenase inhibitory activities	[42]
	caffeoyl-beta-D-glucopyranoside	C_14_H_18_O_9_	antioxidant effect	[41]
	1,3-dihydroxy-2-tetracosanoylamino -4-(E)-nonadecene	C_43_H_80_NO_3_	antioxidant effect	[41]
	ranuncoside	C_22_H_11_O_7_	antioxidant effect	[42]
*Ranunculus ternatus* Thunb.	n-butyl-β-D-fructofuranoside	C_10_H_20_O_6_	inhibitory effect of multidrug-resistant tuberculosis	[48]

**Table 2:** Therapeutic activity of *Ranunculus* species.

*Ranunculus* Species	Therapeutic Activity	Therapeutic Indications	Source	Ref.
*Ranunculus sceleratus* Linn.	Anti-inflammatory	Inhibits nitrite accumulation in macrophage	ethanolic extract of whole plant	[7,31]
	Hepatoprotective (treatment of cholestasis hepatitis)	Improves serum hepatic enzyme activity and hepatic pathologic changes in cholestatic rats	fresh *R. sceleratus* of whole plant	[55]
	Antihypertensive	Inhibits angiotensin converting enzyme (ACE) Involes muscarinic receptor, ganglionic block, and NO	aqueous fraction of aerial parts and roots	[28,54]
	Antiviral	Inhibits hepatitis B virus replication	isolated compounds of whole plant	[32]
*Ranunculus japonicus* Thunb.	Antirheumatoid arthritis	Inhibits migration capacity of rheumatoid arthritis fibroblast-like synoviocytes	methanolic extract of whole plant	[50]
	Antimalarial	Inhibits parasite growth in *Plasmodium falciparum* and *P. berghei* improve hepatic and renal parameters	ethanolic extract of whole plant	[56]
	Antihypertrophic	Suppresses elevated expression of the ANP, BNP, and beta-MHC inhibits up-regulation of [Ca^2+^] *i*	total glycosides of whole plant	[57]
	Protective effect of myocardial ischemic- reperfusion injury	Improves heart function indexes Reduces the area of myocardial infarction	total glycosides of whole plant	[58]
	Antihypertensive	Decreases blood pressure and reduces calcium ions level in cells	total glycosides of whole plant	[36]
*Ranunculus muricatus* Linn.	Antioxidant	Scavenges the DPPH free radical Inhibits lipoxygenase and urease enzyme activity	methanolic extract of whole plant ethyl acetate fraction of whole plant	[41,42]
	Anticarcinogenic	Shows cytotoxic activity to cancer cells Inhibits acetylcholinesterase and alpha glucosidase	ethanolic extract of whole plant	[43]
	Anti-inflammatory Analgesic	Inhibits paw edema, paw licking and abdominal constrictions/stretching of hind limbs	methanolic extract of whole plant	[59]
	Cardiotonic (cardiovascular)	Increases perfusion pression and force of contraction Increases heart rate	methanolic extract of whole plant	[60]
*Ranunculus ternatus* Thunb.	Anticarcinogenic	Induces cell death depending on caspase-7	ethyl acetate extract of whole plant	[61]
	Antibacterial	Shows inhibitory activity against *Mycobacterium tuberculosis* Inhibits multidrug-resistant tuberculosis	ethanolic extract of roots	[48]
*Ranunculus arvensis* Linn.	Antioxidant	Shows antioxidant activity in DPPH free radical scavenging assay	methanolic extract of whole plant	[49]
	Anticarcinogenic	Induces cell death	aqueous and methanolic extract of whole plant	[62]
*Ranunculus diffusus* DC.	Anti-inflammatory	Suppresses NF-kB signaling targeting Src and Syk	methanolic extract of aerial parts	[63,64]
*Ranunculus sardous* Crantz.	Anti-inflammatory	Inhibits nitrite accumulation in macrophage	ethanolic extract from aerial and root parts	[35]
*Ranunculus ficaria* Linn.	Anti-inflammatory	Inhibits nitrite accumulation in macrophage	ethanolic extract from aerial and root parts	[33,35]
*Ranunculus hyperboreus* Rotlb.	Anti-inflammatory	Decreases the elevated nitrate amount Regulates the expression and protein levels of inflammation-related enzymes, iNOS and COX-2, and proinflammatory cytokines, TNF-α, IL-1β, and IL-6 Suppresses activation of MAPK pathway	aqueous and methanolic extract of whole plant	[65]
*Ranunculus pedatus* Waldst. & Kitt.	Anti-inflammatory	Inhibits increased capillary permeability induced by acetic-acid	methanolic extract of whole plant	[66]
	Wound healing	Shows fast dermal remodeling and re-epithelization in epidermis Enhances hydroxyproline content	methanolic and aqueous extract of whole plant	
*Ranunculus constantinapolitanus* (DC.) d’Urv	Anti-inflammatory	Inhibits increased capillary permeability induced by acetic-acid	methanolic extract of whole plant	[66]
	Wound healing	Enhances hydroxyproline content	methanolic extract of whole plant	

## Data Availability

Not applicable.
